# Plant-based diets for human health with implications for cardiometabolic health, gut microbiome, and nutritional adequacy

**DOI:** 10.3389/fnut.2026.1830797

**Published:** 2026-04-28

**Authors:** Mansour Alblaji

**Affiliations:** Department of Basic Health Sciences, College of Applied Medical Sciences, Qassim University, Buraydah, Saudi Arabia

**Keywords:** cardiovascular disease, circular food systems, greenhouse gas emissions, gut microbiome, nutritional adequacy, sustainability, vegan nutrition

## Abstract

Plant-based diets have gained considerable interest in recent times due to their perceived health and environmental benefits. However, the term “plant-based diet” refers to a broad range of diets with a wide range of differences in food quality and nutritional content. This article aims to summarize the available evidence regarding the health and environmental impacts of plant-based diets, including new trends and issues. Epidemiological studies, RCTs, and life cycle assessment studies were searched in various scientific databases to investigate the relationship between plant-based diets and various health outcomes or environmental impacts. Available evidence from prospective studies and RCTs suggests that well-planned plant-based diets are associated with a lower risk of cardiometabolic diseases and beneficial alterations in the gut microbiome. Environmental studies have uniformly found lower greenhouse gas emissions, land use, and water use associated with a human diet compared with an animal-based diet; however, the extent of these positive impacts depends on the diet composition. On the other hand, nutrient adequacy such as iron, vitamin B12, omega-3 fatty acids, processing, and socio-economic factors are also important considerations. This review aims at an integrated approach in environmental sustainability viewpoints with health results, comprehensive understanding the relationship between the quality of diet and health outcomes in the context of a circular food system and research priorities in guiding future diets.

## 1 Introduction

The global food system is confronted with the challenge of rising rates of non-communicable diseases, worsening environmental damage that put pressure on the planet’s limited resources. Though various solutions proposed to address these intertwined challenges and one of them is the consumption of plant-based food which has attracted a lot of scientific, policy, and public attention recently (1). In essence, plant-based diets are highly composed of fruits, vegetables, legumes, whole grains, nuts, and seeds. Thus, they are not only a method of improving nutrition but also a comprehensive approach to the health and wellbeing of humans. Over the last 50 years, epidemiological studies have shown a consistent association of plant-based dietary patterns intake with significantly decreased risk of cardiovascular disease, type 2 diabetes, some type of cancers and all-cause mortality (2). Therefore, the concept of population-level preventive medicine effectively supported by plant-centric diets. Gut microbiota, lower levels of inflammation and oxidative stress because of dietary choices, improvement of cardiovascular disease and provision of anti-carcinogenic phytochemicals are among the mechanisms involved in this process (3). In fact, these mechanisms become better understood in recent years, both individually and collectively, thus explaining the range of health benefits that have been recorded in epidemiological and interventional studies (4).

The food systems of the world have a significant impact on the environment. According to the Food and Agriculture Organization of the United Nations, the livestock sector of the food systems of the world contributes approximately 14–15% of the total greenhouse gas emissions of the world. These greenhouse gases are emitted in the form of methane due to enteric fermentation, manure management, feed production, and land use (5, 174, 175). Besides this, it is a significant driver of deforestation, freshwater depletion, soil degradation, and biodiversity loss, all at unprecedented scales. Numerous scientific organizations such as the Intergovernmental Panel on Climate Change (IPCC) and the EAT-Lancet Commission on Food, Planet, and Health, as well as many individual scientists working on sustainability issues, agree that reducing meat consumption and raising the proportion of plant foods in human diets represent major levers of global mitigation of climate change and maintenance of the earth system’s biophysical boundaries (6, 7). Obesity and diabetes, which are estimated to affect roughly 13 and 9% of adults worldwide, respectively, are preventable chronic diseases for which poor nutrition quality is a significant risk factor (8, 9).

Recent studies have focused specifically on either the health implications or the environmental implications of plant-based diets (10, 11). However, some studies tend to consider these two aspects separately and may miss important nuances between different plant-based diets, levels of food processing, and considerations related to nutritional adequacy. Moreover, as a new paradigm of circular food systems is being proposed, which includes considerations of sustainability, reduction of food loss and waste, and nutrient management, there is a lack of studies that have assessed plant-based diets in relation to this new concept of a more sustainable food system. Therefore, this review attempts to provide a summary of the current evidence related to health, nutrition, and environmental implications of plant-based diets while critically evaluating their place within a more sustainable food system.

Nutrition science, environmental life cycle assessment, microbiome research, behavioral science, and food systems analysis were among the fields from which the evidence was drawn to produce, which allows researchers, clinicians, and policymakers to make informed decisions based on transforming potential and challenges of moving towards plant-dominated diets (3). The main proposition is that a plant-based diet, if it is implemented with due consideration for nutritional adequacy, food quality, equitable access, and systemic food system design, can be a major factor in the advancement of the common good and planet health at least until the next few decades (2, 12, 13).

## 2 Plant-based diets

### 2.1 Spectrum of plant-based dietary patterns

Plant-based diets are a continuum rather than a specific dietary pattern, acting as a broad term for various kinds of eating habits, from flexitarian and mediterranean-style eating to vegan and vegetarian approaches. These diets vary considerably from each other in the extent of avoidance of animal products, nutritional content, and health and environmental consequences (14). For instance, vegan diets are characterized by the absence of all animal products, while vegetarian diets contain dairy and/or eggs (15, 16). Flexitarian diets are rich in plant foods with occasional inclusion of animal products, and pescatarian diets contain fish with no inclusion of terrestrial meats (14, 17). Understanding this spectrum enables readers to comprehend the practical consequences for health and sustainability goals. For instance, vegan diets may increase the danger of certain nutrient deficits if not carefully managed, while pescatarian diets might offer benefits like omega-3 fatty acids. These differences are important as nutritional, environmental, and health consequences may vary depending on the specific plant-based diet (Table 1).

**Table 1:** A summary of comparing vegan, vegetarian, flexitarian, pescatarian dietary patterns.

Diet type	Animal products allowed	Key characteristics	Potential nutritional considerations
Vegan	None	Entirely plant foods	B12, iodine, calcium, zinc; required careful planning’s
Vegetarian	Dairy and/or eggs	Excludes meat/fish	Possible lower intake of vitamin B12, iron, omega-3; eggs can reduce the risk
Pescatarian	Fish allowed	Plant-centered with seafood	Generally nutritionally balanced, addition of mercury from fish, omega-3 for enhancement
Flexitarian	Occasional meat	Mostly plant foods	Fewer the deficiencies if well planned. Adequacy depends on the diet balance

Source: Hargreaves et al. (14), Bruns et al. (15), Miketinas and Champagne (16), and Rossi et al. (17).

### 2.2 Quantitative dietary indices

The great variation in plant-based diet is not only of theoretical interest but bears on the real world in important ways, e.g., making it difficult for nutritionists to pronounce from the literature definitively about the health impact of plant-based diets and it sends a mixed message to the general public (18). To measure the extent of compliance with a plant-based diet, a measure called the Plant-Based Diet Index (PDI) was created by Satija et al. (19). Under this index, foods are grouped into three categories:

1. Healthy plant foods: whole grains, fruits, vegetables, nuts, legumes, vegetable oils.
2. Less healthy plant foods: refined grains, sweets, sugary drinks, potatoes.
3. Animal products.

For the total PDI, all plant-based diets have positive dietary component scores, whereas animal products have negative scores. Under the healthful PDI (hPDI), only healthy plant foods have positive scores, whereas animal products and less healthy plant foods have negative scores. Under the unhealthful PDI (uPDI), less healthy plant foods have positive scores. These scores are given to each food component of the diet depending on whether it is more of an animal or plant product, with the latter classified ones getting a positive score and the former a reverse one (20). The overall score reflects the relative contribution of plant versus animal foods, with positive points assigned to plant foods and negative points to animal foods. The hPDI additionally makes a distinction between healthful and less healthful plant-based foods. The former includes whole grains, fruits, vegetables, legumes, nuts, and healthy plant-based oils whereas the latter include refined grains, potatoes, sugar-sweetened beverages and processed snack foods (21, 22). Case study of health benefits will address what are the key pathways through which such eating patterns confer health benefits: in the primary cohorts, engaging in healthful plant foods is the overall effect that eliminates some scatters in association found between diet and health (23). Higher uPDI scores are linked to unfavourable metabolic outcomes, while higher adherence to hPDI has been linked to lower risk of type 2 diabetes and cardiovascular disease. This highlights the significance of differentiating between plant-based diets that are healthy and those that are not. These indicators are important because they help explain discrepancies in observed health outcomes by differentiating diets high in whole plant meals from those dominated by refined plant products.

### 2.3 Whole foods versus ultra-processed plant-based products

Recently from nutritional science, a crucial distinction made regarding whole-food plant diets vs. those consisting of highly processed plant foods, e.g., commercial plant-based meat substitutes, dairy alternatives, and ultra- processed vegan snacks (24). Although the latter may in fact satisfy plant-based classification criteria, their nutritional characteristics vary greatly from those of unprocessed or minimally processed whole-food plant diets, often containing higher levels of sodium, saturated fat from tropical oils such as coconut and palm, refined carbohydrates, and various additives including colorants, emulsifiers, and flavor enhancers whose long-term health effects remain uncertain (25). Research studies performed on large prospective cohorts, like the NutriNet-Santé study, found that a high consumption of ultra-processed foods, like ultra-processed plant foods, is associated with cardiovascular diseases, cancer, and all-cause mortality, even after adjusting for the quality of the diet (26). This group of studies again reiterates that the primary determinants of the health benefits of plant-based diets are the quality, variety, and level of processing of foods, rather than the absence or presence of animal products (27).

Ultra-processed plant-based diets are also found to have higher quantities of sodium content (400-900 mg per serving) as well as saturated fats derived from coconut or palm oil (5-8 g), as opposed to whole food plant-based diets that are found to have low quantities of sodium and high quantities of fiber. Observational studies have also demonstrated that diets containing high levels of ultra-processed foods increase the risk of cardiovascular disease, obesity, and death from any cause, including plant-based ultra-processed foods. According to recent meta-analyses, ultra-processed plant foods are linked to quantifiable health risks: they frequently contain 30–50% more sodium than minimally processed alternatives, and excessive intake is linked to a 20–30% higher risk of all-cause mortality (28). This demonstrates that the processing of foods is an important factor in health outcomes, regardless of whether the food source is derived from plants or animals (25).

### 2.4 Global and cultural dimensions

Many traditional diets around the world are inherently mostly plant-based and. In fact, their origins lie in the relationship to the local bio-ecological environment and the people’s lifestyles (29). For instance, staples such as rice, legumes, starchy vegetables, and seasonal fruits have typically made up the bulk of people diets in many parts of South and Southeast Asia, sub-Saharan Africa, and Latin America while animal-based foods usually considered only occasional treats or festive dishes (30, 31). Once these traditional plant-centric diets consisted of various whole foods and thus these were amongst the most nutritionally complete and healthiest diets described in the world epidemiological studies. However, depending on the variety and availability of local foods, some traditional diets might additionally have limitations, such as the possibility of vitamin deficiencies in particular areas. But these traditional diets are quickly being replaced by modernity and globalization. For instance, refined carbs, packaged snacks, and sugary drinks have replaced pulses, coarse grains, and vegetables in India’s “nutrition transition,” which has led to an increase in the country’s obesity and diabetes rates. Similar to how fast food and ultra-processed imports are gradually replacing traditional diets based on maize and beans in Latin America, urbanization in sub-Saharan Africa has increased reliance on processed staples and fried street meals (32). The worldwide nutrition transition that is currently undergoing rapid displacement of traditional plant-based eating by Westerner-style diet with high level of ultra-processed foods, refined carbohydrates, and animal products is the main driver of non-communicable disease epidemic in low- and middle-income countries (33, 34). Recognizing the existence of many different plant-based dietary traditions culturally means that plant-centered eating is nothing new and in fact, is more of a revival and update of the original, sapient ecological food habits that have supported human communities for a very long time in any environment and cultural setting (12, 30).

## 3 Health impacts of plant-based diets

The effects of plant-based diets seen in every organ system of the human body. These effects reflect the vast and diverse biological mechanisms through which diet interacts with human physiology (35). An epidemiological study in conjunction with mechanistic researches, randomized controlled trials as well as analysis of diet, gene and diet, microbiome interactions have cumulatively built up a robust and continually growing scientific foundation that supports the use of plant-predominant diets for health maintenance and chronic disease prevention throughout the lifespan (36). The evidence for the beneficial effects of plant-based diets on human health is strongest in the case of cardiometabolic and colorectal cancer prevention, while there are still aspects of this effect in other disease areas that are yet to be uncovered (33, 34). The biological processes involved in the impact of diet on health are quite complex and diverse. This complexity arises from the fact that food is a mixture of thousands of bioactive compounds that interact and influence each other as well as the host’s multiple physiological systems; also, health effects of dietary patterns depend upon the combination of these compounds and the genetic background, gut microbiome, and lifestyle habits of the individual (37). The focus on a very limited number of nutrients in the past for a long time slowed down the understanding of such interactions and now the whole field is increasingly turning to the use of analyses of entire-dietary-patterns that had better represent the overall biological effects of food consumption patterns (38). Four major health domains taken into consideration: (a) cardiometabolic health; (b) prevention of cancer; (c) modulation of gut microbiota, and (d) inflammation and oxidative stress (39). Taken together, these four account for the majority of the chronic diseases in high-income and to an increasing extent in low-income population worldwide.

### 3.1 Cardiometabolic health

Plant-based diets can impact cardiometabolic health through various means such as effects on lipid profiles, systemic inflammation reduction, insulin sensitization, and effects on body weight (33, 34). High fiber content in plant-based diets increases satiety and helps manage blood glucose levels through delayed gastric emptying and glucose absorption. Fiber content in plant-based diets can decrease levels of LDL cholesterol through binding to bile acids and increasing excretion. Plant-based diets are rich in potassium, magnesium, nitrates, and polyphenols that help manage blood pressure. Adventist Health Study-2 (n > 96,000), EPIC-Oxford, Nurses’ Health Study and Health Professionals Follow-up Study represent the four large prospective cohort studies that have ensured continuity of this finding. These studies have demonstrated time after time that vegans and vegetarians have much lower risk of ischemic heart disease, hypertension, type 2 diabetes, and cardiovascular mortality overall when compared to omnivores (40). To gain even further insight, pooled meta-analyses of prospective cohort studies have been conducted and the results indicate that, following a vegan diet, the risk of developing ischemic heart disease decreases by approximately 25–30 percent as opposed to vegetarians who experience a 20–25 percent risk decrease albeit slightly less than vegans. The various processes through which such changes happen, are paradoxical (41). Firstly, dietary fiber, plant protein, phytochemicals, and the generally favorable nutrient profile of whole-food plant-based diets all work together in a synergistic manner to serve the completely cardiovascular risk factor profile comprising lifestyle, genetic and medical factors (42).

**Figure 1:**
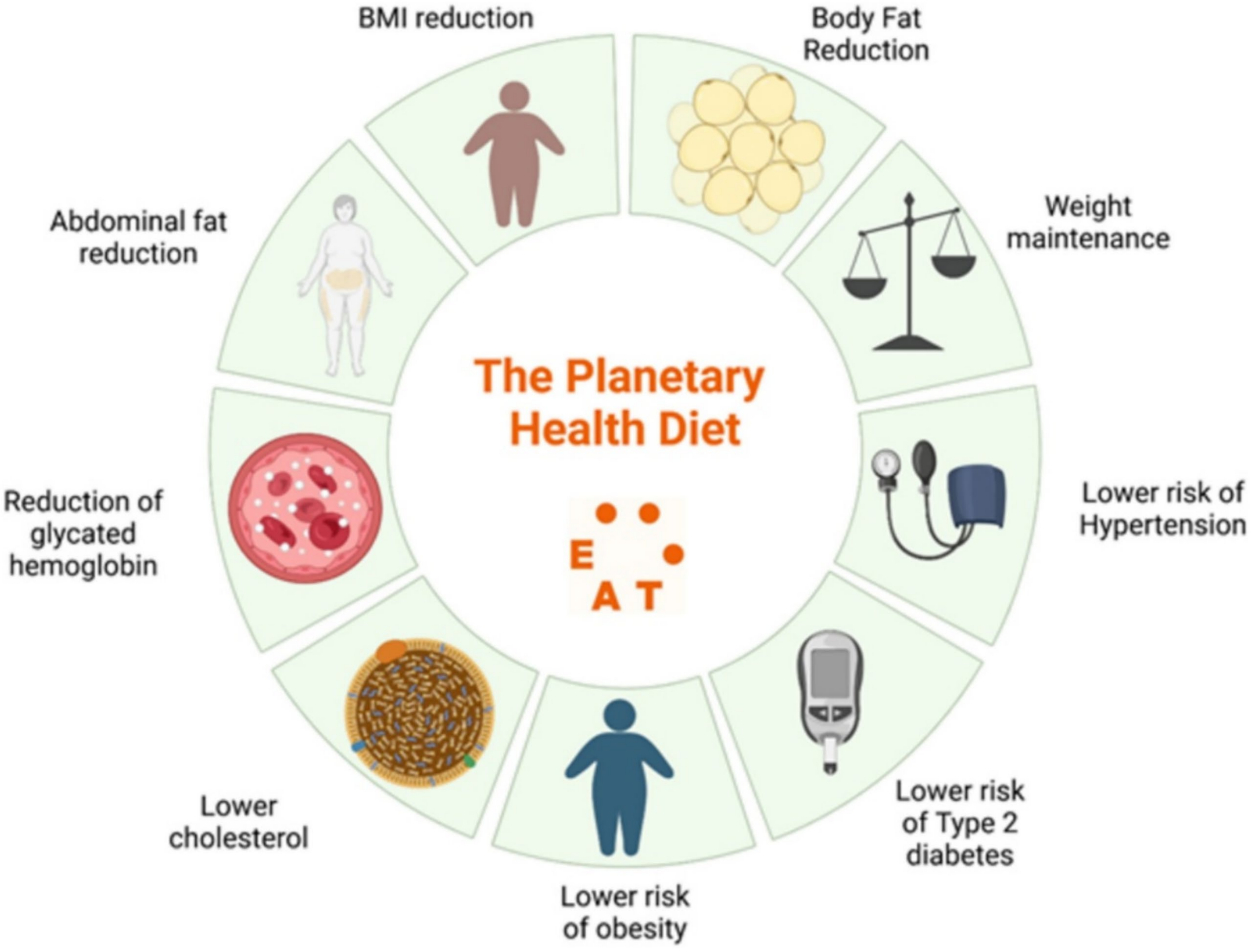
Effects of plant-based diets on human health. Reproduced from Mambrini et al. (162), licensed under CC BY-NC 4.0.

Plant-based diets have been consistently associated with lower body mass index, which considered as one of the major drivers of cardiometabolic risk and additionally with lower incidence of obesity. The effects on body weight brought about by high dietary fiber, which makes one feel full, slows down gastric emptying and reduces postprandial glucose and insulin responses (30). Soluble fiber which can be found in abundance in legumes, oats, barley, and some fruits has the ability to bring down blood cholesterol at a clinically significant level by forming complexes with bile acids that are excreted through feces thus less bile acids are available for cholesterol synthesis in the liver LDL (43). Various clinical trials have shown that well-designed vegan diets can decrease LDL cholesterol by 10–25%, though these reductions are less than those achieved with drug therapy (30). These reductions are lesser than those attained with pharmaceutical therapy, despite the fact that they are clinically significant. High-intensity statins can drop LDL cholesterol by 50% or more, while moderate-intensity statins usually reduce levels by 30–49% (44). Therefore, plant-based diets offer significant supplementary advantages, especially for people looking for non-pharmacological methods or further lifestyle-based risk reduction; but, in high-risk patients, they should be viewed as an addition to medication therapy rather than its replacement. Regulating of blood pressure offers one more place of benefit: plant nitrates from leafy greens and beets convert to nitric oxide, which causes blood vessels to relax and widen, meanwhile the high potassium and magnesium content of plant foods lessen the effect of sodium on blood pressure and stiffening of arteries (45). Both observational and interventional studies have demonstrated that plant-based diets improve insulin sensitivity and thus reduce insulin resistance. The Diabetes Prevention Program and several other randomized controlled trials have demonstrated a 20 to 50 percent reduction in the incidence of type 2 diabetes in plant-based dietary intervention groups relative to controls (46) (Figure 1).

### 3.2 Cancer prevention

There is a sizable and biologically plausible body of research connecting plant-based dietary patterns to lower cancer risk, though in a more subtle and cancer-site specific manner than the cardiometabolic evidence (47). The World Cancer Research Fund and the American Institute for Cancer Research through their detailed worldwide reviews revealed high fiber, vegetable and fruit intake as likely protective factors against several cancers, whereas red and processed meat consumption is classified as a definite (Group 1) carcinogen for colorectal cancer and a probable (Group 2A) carcinogen for stomach cancer (48, 49). The colorectal cancer evidence is strongest: dietary fiber, resistant starch, and short-chain fatty acids, especially butyrate, produced via microbial fermentation of plant polysaccharides in the colon synergistically protect the colonic epithelium by reducing fecal transit time, diluting and adsorbing potential carcinogens, maintaining mucosal barrier integrity, activating tumor suppressor genes, and promoting apoptosis in pre-malignant colonocytes (50, 51).

Large cohort studies that have been conducted, including the Adventist Health Study-2 and the EPIC-Oxford study, have indicated that the incidence of certain types of cancer, including colorectal and gastrointestinal cancers, is low in individuals who follow plant-predominant diets. A meta-analysis of the effects of the consumption of dietary fiber, fruits, and vegetables on the risk of colorectal cancer has indicated that the risk of colorectal cancer is low in individuals who consume more of these food items. A higher intake of red and processed meat has also been linked to an increased risk of cancer (51).

Plant foods contain an impressive array of anti-cancer phytochemicals. Cruciferous vegetables and their bioactive compounds broccoli, cauliflower, Brussels sprouts, kale, and watercress contain glucosinolates that the microflora and enzymes convert to isothiocyanates sulforaphane and indole-3-carbinol. These compounds increase levels of phase II detoxifying enzymes, inhibit the activities of cytochrome P450 enzymes that activate carcinogens, modulate the Nrf2 pathway, and initiate apoptosis and cell cycle arrest in various cancer cell lines (52). Lycopene, the pigment of ripe tomatoes, has been related to the lowered risk of prostate cancer in many prospective studies, especially from cooked tomato products that increase its bioavailability (53). Curcumin, quercetin, resveratrol, and EGCG from turmeric, onions, grapes, and green tea leaves, respectively; display anti-proliferative, anti-angiogenic, and pro-apoptotic activities in various cell line and animal model systems. Multivariate analysis of the Adventist Health Study-2 cohort revealed that vegans had 8 to 16 percent lower incidence of total cancers compared to non-vegetarians. Gastrointestinal and hormone-sensitive cancers were most common (54).

### 3.3 Gut microbiome modulation

The gut microbiome, a complex community of trillions of microorganisms living in the human digestive tract recently recognized as one of the main mediators of the beneficial effects of plant-based dietary patterns (55). The generous amount of dietary fiber in plant-based diets serves as diverse fermentable substrates that lead to enhanced microbial taxonomic diversity, a generally agreed-upon indicator of gut health, and the selective enrichment of fiber-fermenting bacterial taxa such as *Faecalibacterium prausnitzii*, *Roseburia intestinalis*, *Bifidobacterium longum*, *Lactobacillus* species, and members of the *Ruminococcaceae* family, which collectively synthesize short-chain fatty acids, mainly butyrate, propionate, and acetate, as their principal fermentation metabolic products (55, 56). Intervention studies with diet have shown that an increase in the amount of plant fibers and polyphenol-rich foods in the diet rapidly changes the composition of the microbiota in the gut, with a significant increase in beneficial bacteria such as *Faecalibacterium* and *Bifidobacterium*. These bacteria have a positive effect on the production of short-chain fatty acids, especially butyrate (50).

**Figure 2:**
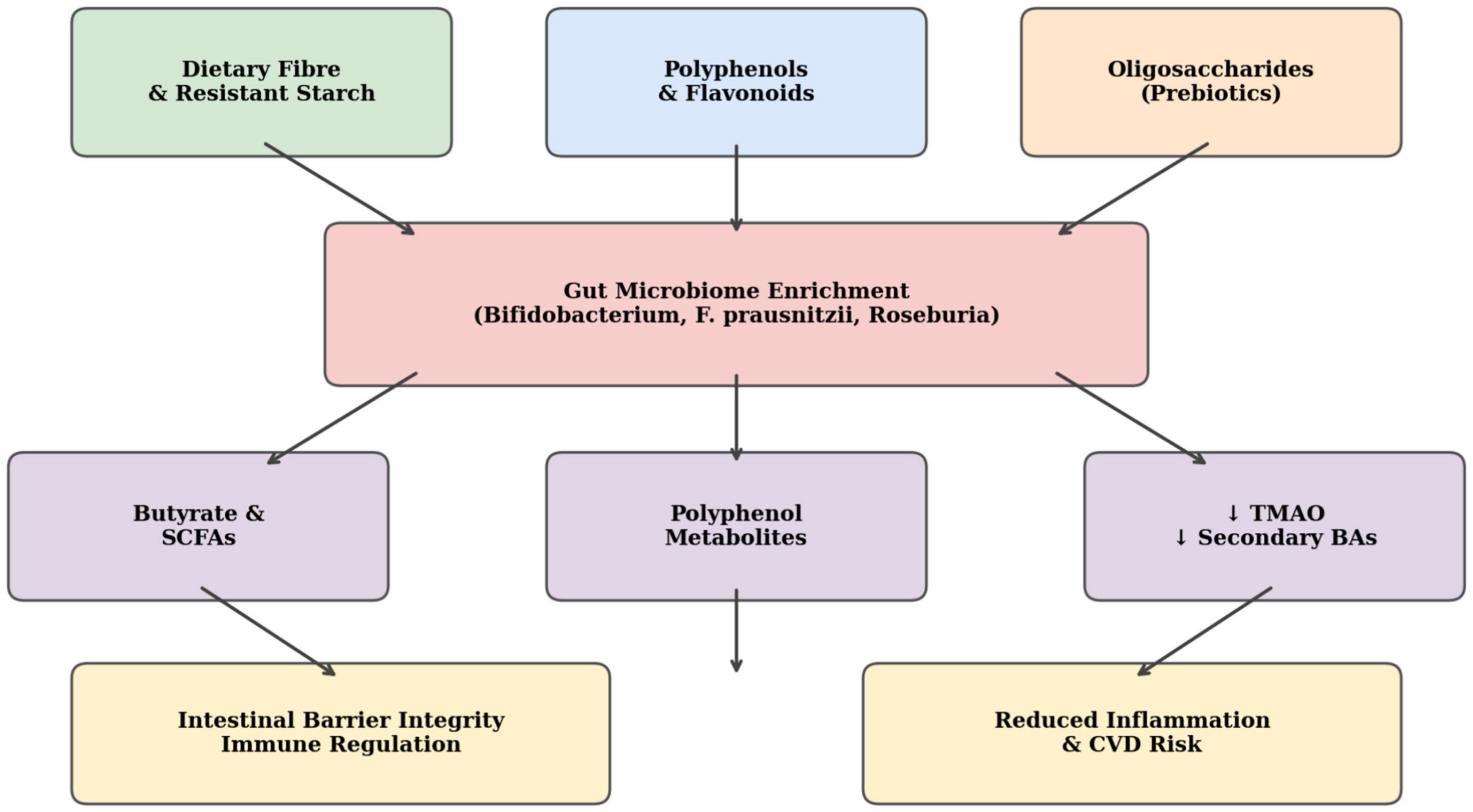
Mechanistic pathways of gut microbiome modulation by plant-based dietary components and their downstream effects on intestinal and systemic health. (SCFAs, Short-chain fatty acids; TMAO, Trimethylamine N-oxide; BAs, Bile acids). Adapted from Mao et al. (163), licensed under CC BY 4.0.

Butyrate is the main energy source for colonocytes and it performs several important functions such as maintaining the integrity of the intestinal epithelial barrier, regulating mucosal immune homeostasis, suppressing colonic NF-κB-driven inflammation, inducing histone deacetylase inhibition, histone acetylation, gene expression changes, epigenetic gene regulation, and promoting apoptosis in pre-neoplastic colonocytes (50). Significantly, low levels of butyrate-producing bacteria and levels of fecal butyrate have been consistently found in patients suffering from inflammatory bowel disease, colorectal cancer, obesity, and type 2 diabetes. Thus, it suggests that dysbiosis of the gut microbiota might play an important role in the potential link between diet and disease. Polyphenols present in plant-based diets also play an important role in modulating the gut microbiota. Metabolism of these compounds by Lactobacillus, Bifidobacterium, and Bacteroides species produces urolithins, equol, and other compounds that have anti-inflammatory, estrogenic, and anti-proliferative properties (57). Additionally, the consumption of plant-based foods has been seen to dramatically decrease the production of potentially harmful microbial metabolites such as trimethylamine N-oxide (TMAO), which is produced from dietary carnitine and choline by certain bacteria and is recognized as an important predictor and cause of atherosclerosis and cardiovascular events in prospective studies (58, 59). Most of research highlights the enhancement of advantageous butyrate-producing species, despite few studies reporting decreases in certain taxa, such as Bacteroides, with high-fiber diets. Because of this, while recognizing that not all microbial alterations are always advantageous, discussion focuses on the positive changes that are most frequently reported. It should also be noted that individual variability in microbiome responses are driven by genetics, geography, and baseline microbiota composition. This could help to explain some of the variations in study results. Figure 2 shows the main mechanisms by which various compounds of plant-based diets regulate the composition of the gut microbiota and health outcomes.

### 3.4 Inflammation and oxidative stress

It is understood that chronic low-grade systemic inflammation and oxidative stress are the primary linkage mechanisms between a person’s diet and the development of cardiovascular disease, type 2 diabetes, neurodegenerative disorders, and many cancers (60). Compared to other diets, plant-based diets consume have been figured to be strongly anti-inflammatory and antioxidant which is due to multiple parts of the diet working together to affect several complementary molecular pathways (61–63). The epidemiological studies also confirm this by showing that vegetarians and vegans have statistically significant lower levels of circulating inflammatory markers such as high-sensitivity C-reactive protein, interleukin-6, interleukin-1β, tumor necrosis factor-alpha, and fibrinogen as compared to omnivores even when adjusted for body mass index, physical activity, and other lifestyle confounders (33, 34). However, not all research consistently shows decreases in inflammatory markers, and variables including lifestyle, genetics, and BMI may affect outcomes, which should be taken into account when interpreting results.

Multiple components of the diet are responsible for the anti-inflammatory effects of plant-based diets. One of the mechanisms is through dietary fiber and its main fermentation product, butyrate, NF-κB signaling (a key transcriptional regulator of inflammatory gene expression) suppressed by lowering IκB kinase activity and increasing HDAC inhibition (64). Polyphenols and flavonoids such as quercetin, kaempferol, luteolin, and apigenin not only inhibit pro-inflammatory cyclooxygenase-2 and lipoxygenase enzyme activities, but they also decrease NF-κB and AP-1 transcription factor activation, and regulate the NLRP3 inflammasome pathway (65).

Alpha-linolenic acid found in flaxseeds, chia seeds, hemp seeds, and walnuts is used for the production of anti-inflammatory resolvins and protectins through cyclooxygenase and 15-lipoxygenase pathways, which is why it is considered an excellent contributor to the dietary omega-6 to omega-3 fatty acid ratio that deviates from the highly pro-inflammatory values characteristic of typical western diets (66). Regarding oxidative stress, plant-based diets are loaded with dietary antioxidants such as vitamin C, vitamin E, beta-carotene, lycopene, lutein, zeaxanthin, selenium, and manganese cofactors of superoxide dismutase and glutathione peroxidase that nourish the body’s antioxidant defense system (67). Sulforaphane from cruciferous vegetables, curcumin from turmeric, and cinnamaldehyde from cinnamon activate the Nrf2, Keap1 pathway, thereby increasing cytoprotective enzyme expression, which yields long-lasting, transcriptionally mediated antioxidant protection that significantly scales up the direct radical-scavenging activities of individual dietary antioxidants (68–70) (Table 2).

**Table 2:** Key nutrients of concern in plant-based diets: sources, bioavailability, and recommendations.

Nutrient	Plant sources	Bioavailability challenge	Recommended strategy	Life stages of concern	References
Vitamin B12	Fortified foods, nutritional yeast	Absent in unfortified plant foods; deficiency may lead to neurological damage	Daily B12 supplement (cyanocobalamin); consume fortified plant milks	All life stages; critical during pregnancy and infancy	Craig (164) and Pawlak et al. (165)
Iron (non-heme)	Legumes, tofu, spinach, seeds, fortified cereals	Absorption inhibited by phytates and polyphenols; lower absorption than heme iron	Pair with vitamin C; soak/sprout legumes; avoid tea or coffee with meals	Menstruating women; pregnancy; growing children	Hurrell and Egli (166) and Gibson et al. (167)
Calcium	Kale, bok choy, calcium-set tofu, fortified plant milks, almonds	Oxalates reduce absorption in some greens such as spinach	Choose low-oxalate greens; consume fortified plant milks regularly	Children, adolescents, older adults, pregnant women	Weaver and Heaney (168) and Mangels et al. (169)
Omega-3 (EPA/DHA)	ALA in flaxseed, chia seeds, walnuts; algae-derived EPA/DHA	Limited conversion of ALA to DHA/EPA	Use algae-derived omega-3 supplements	Pregnancy, breastfeeding, infancy, older adults	Davis and Kris-Etherton (170)
Zinc	Legumes, whole grains, nuts, seeds, fortified cereals	Phytates reduce zinc bioavailability	Use soaking, sprouting, fermentation; slightly higher intake may be required	Children; pregnant and lactating women; elderly	Gibson (171)
Iodine	Seaweed, iodized salt, fortified plant milks	Iodine levels in seaweed vary greatly	Use iodized salt or iodine supplements when dairy is excluded	Pregnant and lactating women; growing children	Bath and Rayman (172)
Vitamin D	Sun-dried mushrooms, fortified foods, sunlight exposure	Limited dietary sources; sunlight exposure may be insufficient	Vitamin D supplementation; monitor serum 25(OH)D	All populations; particularly older adults and those in northern latitudes	Holick (173)

ALA, alpha-linolenic acid; EPA, eicosapentaenoic acid; DHA, docosahexaenoic acid; SCFAs, short-chain fatty acids.

## 4 Nutritional adequacy and potential risks

### 4.1 Vitamin B12 risk

Vitamin B12 (cobalamin) is the nutrient deficiency that most clinically concerns people who strictly follow plant-based or vegan diets. Certain bacteria and archaea exclusively make this vitamin, and nutritionally significant levels only found in animal-derived foods, such as meat, fish, poultry, dairy, and eggs, as well as in B12-producing microorganisms used in food fermentation (71). Although there are some plant foods like certain seaweeds and fermented soy products, which contain B12 analogues, these are mostly non-functional pseudo-B12 forms that do not satisfy human metabolism and may even, in some cases, hinder the absorption of the active cyanocobalamin and methylcobalamin forms (72). These latter forms are very important for adequate B12 status, which, in turn, is necessary for the methylation cycle, which is the basis of DNA synthesis and repair, myelin synthesis, and neurological function, as well as for the conversion of homocysteine to methionine. Lack of these leads to megaloblastic anaemia, subacute combined degeneration of the spinal cord, peripheral neuropathy, cognitive impairment, and elevated homocysteine, a cardiovascular risk factor independently, in the presence of neurological damage may become irreversible, even in the absence of anemia, due to folate masking hematological symptoms (71). Other nutrients that deserve attention in plant-based diets include vitamin B12, iron, iodine, calcium, zinc, and long-chain omega-3 fatty acids. Vitamin B12 is absent in plant-based diets. Vegans should therefore consider vitamin B12-fortified foods or supplements. There is conflicting evidence regarding the health of the bones. Cohort studies have indicated that vegans might be at slightly higher risk of fractures due to low calcium and protein intake. However, adequate calcium intake and vitamin D status may counteract the risk (73).

### 4.2 Iron, calcium, zinc, and bone mineral density

Iron nutrition in plant-based diets presents a bioavailability challenge rooted in the fundamental distinction between heme and non-heme iron forms (74). Plant foods including legumes, tofu, tempeh, pumpkin seeds, quinoa, dark leafy greens, and iron-fortified cereals contain significant quantities of non-heme iron, but this form is substantially less bioavailable than heme iron due to competitive inhibition by dietary phytates in whole grains and legumes, polyphenols in tea, coffee, and certain vegetables, and dietary calcium (75). Bioavailability of non-heme iron ranges from approximately 2–20 percent under typical dietary conditions, compared to 15 to 35 percent for heme iron (76). Despite lower bioavailability, vegetarians and vegans who consume diverse iron-rich plant foods can maintain adequate iron status; iron deficiency anaemia rates are not significantly elevated in well-nourished populations with diverse diets in well-planned plant-based diets in well-nourished populations, though serum ferritin concentrations are generally lower (77). Calcium adequacy without dairy requires intentional dietary planning emphasizing low-oxalate calcium-rich plant foods including kale, bok choy, Chinese broccoli, calcium-set tofu, edamame, fortified plant milks, almonds, and tahini, alongside adequate vitamin D and physical activity to support bone mineral density. Zinc bioavailability from plant foods reduced by phytate chelation to a degree similar to iron; practical mitigation strategies including soaking, sprouting, and fermenting legumes and whole grains can reduce phytate content by up to 50 to 90 percent and meaningfully improve zinc absorption (11). Meta-analyses report modestly lower bone mineral density at the hip and lumbar spine in vegans compared to omnivores, though whether this translates to clinically significant fracture risk elevation with adequate calcium, vitamin D, and physical activity remains under active investigation (78).

### 4.3 Omega-3 fatty acids, iodine, and vitamin D

Long-chain omega-3 fatty acids, namely eicosapentaenoic acid (EPA) and docosahexaenoic acid (DHA), are vital to cardiovascular health, brain development and function, alleviation of inflammation, and vision (79). The primary dietary sources in omnivore diets are marine fish and other seafood; plants only contain alpha-linolenic acid (ALA), whose natural conversion in the body to EPA and DHA is limited due to competition with linoleic acid for the desaturase enzyme, and, therefore, the conversion of ALA to EPA and DHA is generally below 10 and 1 percent, respectively (80). Nutritional surveys continually show low levels of DHA in the blood of vegans, which could be especially concerning to infants’ neurodevelopment and older people’s cognitive function. Supplements with algae-based EPA and DHA, derived from microalgae, which form the original source of marine omega-3 fatty acids, provide vegan-friendly, equivalently nutritious long-chain omega-3 fatty acids and, hence, are highly advised in particular during pregnancy and when breastfeeding (10). Iodine deficiency is a known issue in those who exclude both dairy and seafood from the diet because, in many western countries, dairy products are the main dietary source of iodine, not only naturally but also due to the contamination from iodine-containing teat dips and cattle feed supplements; moreover, besides seaweed, plant foods generally contain very small amounts of iodine (81). Most people get their vitamin D primarily through the skin when ultraviolet-B rays from the sun convert a precursor in the skin to vitamin D; however, this method is inadequate in the winter months at high latitudes, for dark-skinned people and people with little or no exposure to the sun, etc. (82). Supplementation with vitamin D2 or vegan-friendly vitamin D3 (extracted from lichen) is essential to maintain serum 25-hydroxyvitamin D levels at or above the sufficiency level of 50 nmol/L since only a handful of plant foods naturally contain vitamin D (83).

### 4.4 Achieving nutritional completeness across the life cycle

**Figure 3:**
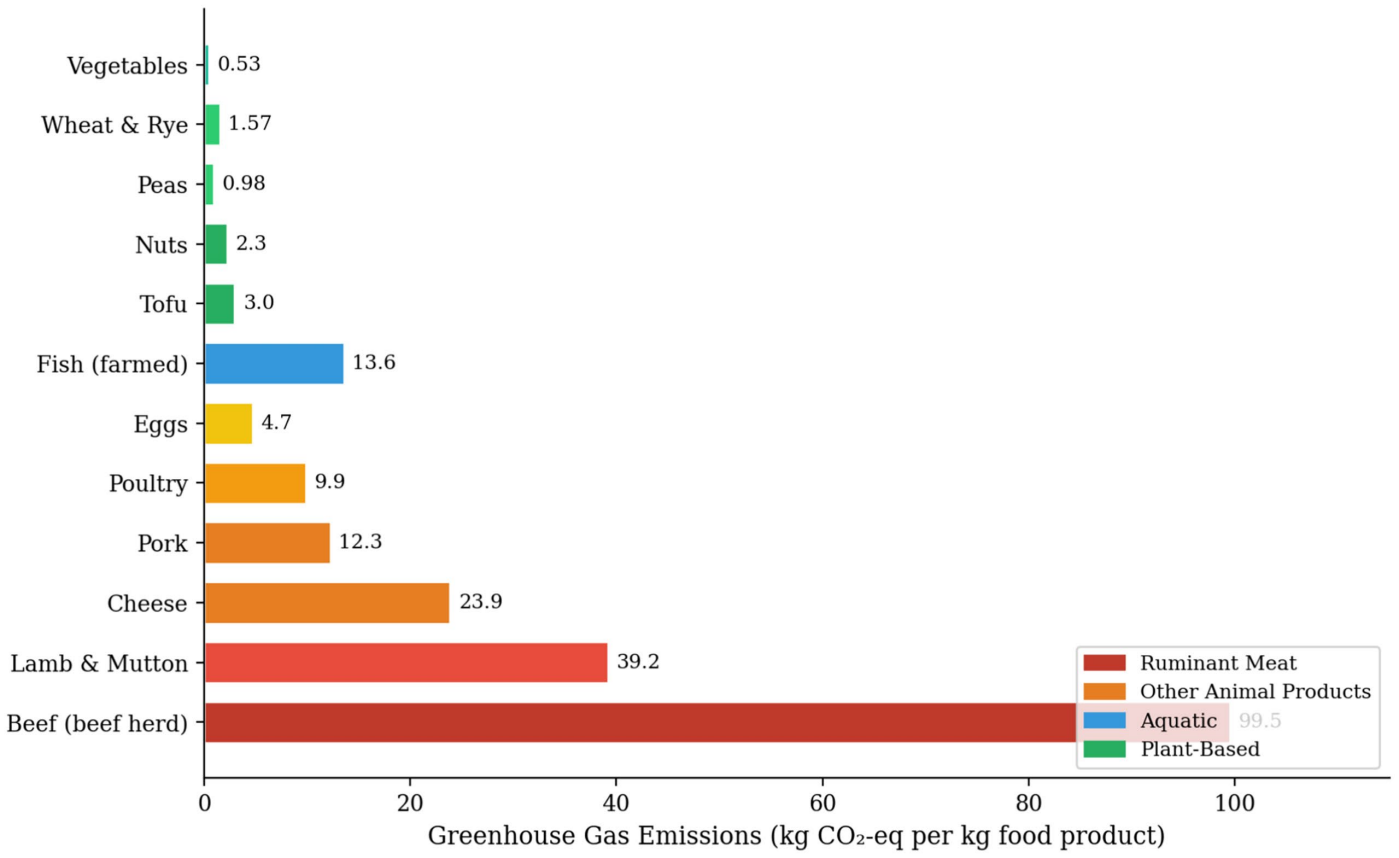
Greenhouse gas emissions (kg CO₂-equivalent per kg food product) across selected food categories, illustrating the marked disparity between animal-based and plant-based foods. Reproduced from Poore and Nemecek (88), with permission from AAAS.

Indeed, nutritional disorders do not have to be the inevitable result of plant-based dietary patterns, even though they are the major obstacle to such patterns, but can see as the lack of good dietary planning, a well-functioning food fortification program, and a system of appropriate and targeted supplementation (11). Nutritional position statements of the Academy of Nutrition and Dietetics, Dietitians of Canada, and the British Dietetic Association along with research articles worldwide, clearly state that properly planned vegan diets are nutritionally sufficient and beneficial to health at any human life cycle’s stage including pregnancy, lactation, infancy, childhood, adolescence, adulthood, and older age (84). The most important factor for nutritional sufficiency in plant-based diets is dietary diversity, i.e., the intake of a wide range and variety of whole plant foods including legumes, whole grains, different vegetables, fruits, nuts, seeds, and unprocessed/ minimally processed plant-based products, instead of the dependence only on a limited number of staple foods (85). Tracking the nutritional status of an individual through dietary recall, food frequency questionnaire analysis, and a focused laboratory check-up of B12, iron, vitamin D, iodine, and omega-3 can help with the early spotting of nutritional gaps and the subsequent formulation of the most fitting dietary advice (10). It is the joint responsibility of healthcare providers, registered dietitians, and public health policymakers to make sure that sound scientific guidance on nutritional adequacy in plant-based diets easily accessible to people of all socioeconomic backgrounds, and that food fortification programs, product labelling standards, and nutrition education materials sufficiently back up the recognition of plant-based dietary patterns as a health-promoting and thus legitimate eating pattern within nation dietary guidelines and clinical practice frameworks (86) (Figure 3).

## 5 Environmental impacts of plant-based diets

### 5.1 Greenhouse gas emissions and climate mitigation potential

The food system, overall estimated to be responsible for 26 to 34 percent of global anthropogenic greenhouse gas emissions. This takes into account the entire supply chain from land use change, through processing, packaging, transportation, retail, and consumer food waste (87). Various studies on life cycle assessment have shown that, on average, foods derived from plants tend to be more environmentally friendly in comparison to foods derived from animals, considering greenhouse gas emissions, land use, and water consumption. For example, beef products tend to produce more than 20–60 kg CO_2_ per kilogram, while most types of legumes tend to produce less than 2 kg CO_2_ per kilogram. However, dietary changes towards more plant-based diets in the global context must be treated with caution (88). The beef from beef herds is responsible for roughly 99.5 kg CO₂-equivalent per kilogram of product that is, more than 60 times the greenhouse gas intensity of legumes, which is mostly due to enteric methane fermentation in ruminant digestive systems, nitrous oxide emissions from manure management and feed crop fertilization, and the carbon opportunity cost of land use (2). Dietary modelling studies of shifts, including the analysis of Springmann (2) indicate that a change to plant-based diets worldwide could help to cut food-related greenhouse gas emissions by 49 to 63 percent by 2050, thus offering one of the single largest mitigation opportunities that the whole economy can provide (89). These forecasts are pertinent to the national climate commitments under the Paris Agreement which focus mainly on the energy and transport sectors and hardly consider the food system’s transformative mitigation potential (90). However, while thinking about global transitions, it is important to recognize that regional variations in agricultural techniques, cultural dietary preferences, and economic viability present obstacles to significant changes.

### 5.2 Land use, deforestation and biodiversity conservation

Animal agriculture, including the land for grazing and growing crops for animal feed, mostly soy and corn, covers about 77 percent of the world’s agricultural land but only accounts for 18 percent of the world’s total calories (91). The extreme land inefficiency is the main reason animal agriculture is a major driver of tropical deforestation, mainly in the Amazon rainforest, Cerrado savanna and Southeast Asia, where natural ecosystems are destroyed on a large scale to make pasture and grow feed crops with biodiversity loss, carbon (88, 92) storage, indigenous communities impact going largely irreversible and devastating. Life cycle assessments show consistently the huge difference in land use efficiency between animal and plant-based diets. For instance, making 100 grams of protein from beef requires about 164 square meters of land, while for chicken it is 7 square meters, 2.2 square meters for tofu, 1.4 square meters for lentils, and less than 1 square meter for peas (93). Food system modelling by Poore and Nemecek shows that, if people worldwide stopped eating animal products, the about 3.1 billion hectares of agricultural land that are currently used for this purpose could be given back to nature or used for regenerative farming, which would bring huge benefits to biodiversity conservation, carbon sequestration in the atmosphere, the freshwater cycle, and the restoration of soil health (94). Such land liberations are an essential prerequisite for the global biodiversity goals, also those of the Kunming-Montreal Global Biodiversity Framework of the Convention on Biological Diversity, which sets the target of protecting 30 percent of terrestrial and marine ecosystems by 2030 (95–97).

### 5.3 Water use and freshwater system protection

Approximately 70 percent of global freshwater withdrawals is used for agriculture and animal product supply chains that comprise direct animal water use and feed crop irrigation that requires a lot of water are responsible for a disproportionately large share of this footprint (98, 99). A buffalo’s virtual water content is 15,400 liters per kilogram of product, in contrast to 1,827 liters for wheat, 1,250 liters for lentils, and 322 liters for vegetables (100). The whole amount of water used in production, including irrigation, processing, and other inputs, is referred to as virtual water content. The water demands of livestock and feed crop agriculture place severe pressure on depleted aquifer systems and surface water resources already stressed by climate change-driven hydrological variability in water-scarce regions such as the western United States, northern India, the Middle East, and sub-Saharan Africa (101). Reactive nitrogen and phosphorus from livestock manure and synthetic fertilizer applications to feed crops contribute, besides the amount of water used, to one of the major problems of water quality that is eutrophication of freshwater leading to oxygen depletion, foam formation, harmful algae, coastal dead zones; in other words, the Gulf of Mexico hypoxic zone caused mainly by agricultural nutrient runoff from Mississippi River basin is a case in point (102). Changing diets toward mainly plant foods can greatly reduce water consumption and nutrient pollution in water bodies (both fresh and marine), but only if the different water needs of various plant foods are taken into account: for example, almonds, avocados, and asparagus have much higher water footprints than most other plant foods because they are typically grown in arid regions whose production requires irrigation (103).

### 5.4 Soil health, carbon sequestration, and regenerative agriculture

The effects of plant-based diets on soils are complex and dependent on the wider agroecological situation. Industrial monoculture farming that grows crops for both animal feed and plant-based human foods, if done without integrating regenerative management interventions, deteriorates land resources through soil compaction, erosion, salinization, nutrient depletion, and lowering of soil microbial and faunal biological diversity (104). However, plant-based food systems tend to have a structure that is more compatible with the implementation of regenerative agriculture as opposed to animal-based food systems (12). Crop rotation practices that use legumes for nitrogen fixation through the action of bacteria rhizobia can largely do away with the use of synthetic nitrogen fertilizers and the associated nitrous oxide emissions, i.e., a greenhouse gas that has the effect equivalent of 298 over the span of 100 years (105). Cover cropping, less tilling, planting trees in the fields, and the use of perennial crops are all examples of plant-based production that can increase soil organic carbon by 0.1 to 0.4 percent per year under good conditions, thereby not only helping to sequester carbon and thus mitigate climate change but also improving soil water retention, aggregate stability, and long-term agricultural production (105). Combining different plant-based dietary patterns with a shift to regenerative and agroecological farming systems is, therefore, an all-encompassing and complementary approach for setting up a food system that could provide nutritious food, restore the environment, be resilient to climate change, and ensure feed security globally at a sustainable level for present and future generations (106). Table 3 lists the environmental footprints in a quantitative manner comparing selected plant- and animal-based food categories.

**Table 3:** Comparative environmental footprints of selected plant-based and animal-based foods.

Food item	GHG (kg CO₂-eq/kg)	Land use (m^2^/100g protein)	Water use (L/kg)	Eutrophication (g PO₄-eq/kg)	Dietary category
Beef (beef herd)	99.5	164.0	15,415	365.0	Animal-based (Ruminant)
Lamb and Mutton	39.2	185.0	10,412	97.0	Animal-based (Ruminant)
Pork	12.3	11.0	5,988	76.0	Animal-based (Monogastric)
Chicken	9.9	7.1	4,325	48.7	Animal-based (Poultry)
Eggs	4.7	5.7	3,265	21.8	Animal-based (Eggs)
Tofu	3.0	2.2	2,145	6.0	Plant-based (Legume-derived)
Lentils	0.9	1.4	1,250	3.6	Plant-based (Legume)
Wheat and Rye	1.6	2.8	1,827	7.4	Plant-based (Cereal grain)
Nuts	2.3	3.5	4,134	2.0	Plant-based (Tree nuts)
Vegetables (avg)	0.5	0.8	322	1.1	Plant-based (Vegetables)

Reproduced from Poore and Nemecek (88), with permission from AAAS; GHG, greenhouse gas; PO₄, phosphate equivalent. Green shading = plant-based diets; Red shading = animal-based foods.

## 6 Plant-based diets within circular food systems

The circular food system concept highlights the importance of the efficient use of biological resources, food wastage reduction, and nutrient recycling. Plant-based diets may contribute to the development of circular food systems by eliminating the need for resource-intensive animal production and enabling the allocation of arable land to food production. However, animal production may contribute to circular food systems by using food wastage that is not fit for human consumption (107). Circular food system is a model that recounts the old linear form of the take-make-waste model adopting instead reduction of resource consumption, recycling of nutrients, reduced food loss and regenerating ecosystems (108). Plant-based diets, which are based on cereal, pulses, fruits, vegetables, nuts, and seeds, are highly congruent with these principles since plant-based products are typically less demanding in natural resources, including land, water, and energy, than animal-based products. Resource efficiency is increased by focusing on crops directly consumed by humans instead of turning them into livestock feed and general environmental pressure is lowered (12). Nutrient cycling is an important component in circular systems. The food waste and the crop residues composted or converted into biofertilizers, which restore the soil with vital nutrients resulting in better health of the soil (109). Leguminous foods like lentils and chickpeas help in fixing nitrogen in nature thus decreasing fertilizers that are synthetically produced which in turn reduces greenhouse gases. Moreover, the processing industries of plants produce by-products such as bran, fruit pomace, and oil-seeds cakes, which could be, valorized in terms of functional foods, dietary fiber sources, or bioenergy sources so that a minimum waste generated and the highest value extracted (110). Plant-based diets also found to favor biodiversity and soil regeneration by crop rotation, intercropping and agroecology practices that enhance resilience to climate change. However, if monocultures like wheat or soy are not managed responsibly, biodiversity may be threatened, underscoring the necessity for varied cropping techniques. Circular plant-based systems have economic opportunities to provide the local food networks, small-scale farmers, and green innovation (111). Nevertheless, effective implementation needs the supportive policies, the awareness of the consumers, waste recovery infrastructure, and the equal availability of the nutritious vegetable products. Altogether, the incorporation of plant-based diets into the circular food systems provides a comprehensive solution direction to sustainable food security, environmental protection, and resilience of global food systems in the long term (12, 112, 113).

### 6.1 Circular economy principles applied to food systems

The circular economy model, as developed mainly by the Ellen MacArthur Foundation and based on older concepts of industrial ecology, biomimicry and ecological economics, radically separation itself from the dominant linear “take-make-dispose” model by presenting economic and biological systems not only as means to regenerate resources and waste elimination but as tools for maintaining material and nutrient value in perpetual productive cycles and, in general, going beyond the idea of waste being the inevitable by-product of economic activity (114). If we consider that food systems are probably the largest human materials flow system and that they have the greatest impact on the environment, implementing circular economy at every stage of food systems means that a set of design imperatives are implemented (108). The biological nutrient cycles between food production, consumption, and organic waste management need to be closed; energy and virgin resource inputs per unit of nutritional output need to be minimized through food supply chain efficiency improvements; the biomass and food processing byproducts need to be used in multiple, value-adding applications before their biological decomposition, and soil, water and ecosystems need to be actively regenerated as a prerequisite for sustainable food production (115). Moving towards plant-based diets by full integration into plant-based diets system designing is not only a highly attractive benefit of the dietary transition but actually a basic requirement for circular food systems that are capable of functioning within planetary ecological limits (116).

### 6.2 Trophic efficiency and nutrient cycling

We can consider from a bioenergetics point of view that if humans eat plants directly it will be more efficient in terms of energy and nutrients because we will avoid the inevitable losses that happen when plant calories and nutrients first eaten by animals and then humans consume animal products (2). Second law of thermodynamics states that every time energy transformed, part of it is lost as heat, so the reason conversion of plant calories to animal product calories is so inefficient becomes clear: animals only convert 10 to 30 percent of the energy in feed into edible products (meat, milk or eggs). Other part of the energy is lost as heat, carbon dioxide and non-edible biomass (117, 118). The retention of nitrogen and phosphorus in animal products is similarly quite limited, only 5 to 40 percent of the nitrogen and phosphorus in feed is found in human-edible meat, milk, and eggs, whereas the rest is excreted as reactive nitrogen compounds including ammonia, nitrous oxide, and nitrate, and phosphorus in manure leading to environmental pollution that goes beyond the farm (119). Therefore, you see with these trophic efficiency losses that land and water resources used to grow animal feed could have produced about 5 to 10 times more nutritional calories for humans if the animals were not present in the system (120). Plant-based dietary practices drastically lower the material and land footprint of food systems by avoiding animal intermediaries in the energy and nutrient cycling, and they also allow food production, consumption, and organic waste recovery systems to be more tightly connected, more efficient, and easier to control with nutrient flows, a must for circular food system design (121).

### 6.3 Organic waste recovery, urban agriculture, and the bioeconomy

In circular food systems, plant-based diets help the system to be more compatible and seamlessly integrated with organic waste recovery and nutrient recycling through the whole value chain of food supply at the local and regional levels (122). Only the plant-based food chains generate organic wastes such as crop residues and stover, food processing by-products like fruit pomace, vegetable trimmings, and oilseed meals, as well as household and food service food waste (123), which are all biological materials that can be converted into compost and liquid digestate enriched in plant nutrients such as nitrogen, phosphorus, and potassium by composting and anaerobic digestion and such biofertilizers can thus close the nutrient cycle between urban consumption and agricultural production (124, 125). There are several modes of urban agriculture, such as rooftop gardens, community gardens, peri-urban farms, and indoor vertical farms (126).

The plant-based diets production system can be more naturally aligned with livestock production at urban scale, therefore the inputs such as reclamation of organic waste from cities can be directly linked as growth inputs for crops by the plant-based system and the distances for food transportation and the emissions can be significantly lowered (2). The concept of a bioeconomy is just taking circular resource recovery one step further by picturing food system residual streams such as lignocellulosic crop residues, fruit pomace, seaweed biomass, and insect frass as material inputs for the successive production of biochemicals, biofuels, biomaterials, and novel food ingredients (127, 128), henceforth after multi-stage valorization layers are formed to maximize the total value extracted from biological resources prior to their final reincorporation into natural cycles through composting or anaerobic digestion (125, 129).

### 6.4 Novel protein technologies and circular food futures

The newly developing food technologies present excellent possibilities to make complete nutritional plant-based protein foods that utilize resources extremely efficiently and have great potential for circular integration (130). Apart from the conventional protein sources, single-cell protein (SCP) production has the potential to save a lot of land and be friendly to the environment due to the microbes such as yeasts, bacteria, microalgae, or filamentous fungi which can be grown on organic waste substrates like agricultural effluents, food processing wastewater, and even atmospheric carbon dioxide and hydrogen, thus allowing for the conversion of low-value waste streams into protein-rich food and feed ingredients, without occupying much land and when powered by renewable energy can have net-positive environmental profiles (48, 49, 131). Mycoprotein is an excellent example of how fermentation-based protein technology can work since it produces protein from wheat-derived glucose which has about 90% less impact on the planet in terms of carbon dioxide emitted and the area of land used to produce one unit of protein compared to chicken production (132). Using genetically engineered microorganisms, precision fermentation can be used to make food proteins, fats, enzymes, vitamins, and flavor compounds with a defined molecular structure, meaning that functionally superior food ingredients such as heme proteins, casein, whey proteins, and vitamin B12 can be produced while land, water and emission footprints are dramatically lowered in comparison to traditional animal or crop based production, which might be able to solve some of the nutritional gaps in the plant-based diets (133). By growing algae we can filter out nutrients from agricultural runoff or aquaculture effluents and at the same time produce biomass rich in complete protein, DHA omega-3 fatty acids, vitamin B12, iodine, and high-value pigments, all these nutrients have been identified as lacking in plant-based diets, hence algae could become a source of these nutrients while the algae cultivation also contributes to protecting surface water quality and achieving circular nutrient management within integrated food-water-energy system frameworks (134, 135).

## 7 Challenges and future research directions

### 7.1 Nutritional challenges

Though, there are many health benefits associated with plant-based dietary patterns, ensuring nutritional adequacy is an important consideration, especially if animal foods are omitted entirely. For example, some nutrients are naturally low or less bioavailable in plant foods. These include vitamin B12, iron, iodine, zinc, calcium, and long-chain omega-3 fatty acids (EPA and DHA), collectively known as vitamin D. Vitamin B12 is particularly concerning because plant foods are unlikely to be reliable sources unless supplemented or fortified. Deficiency in this vitamin has serious health implications if not well addressed because it causes neurological damage and anemia. Iron from plant foods is less bioavailable to the body compared to iron from animal foods because phytates and polyphenols inhibit iron absorption (136–138). These are some of the reasons why dietary planning is crucial to ensure that plant-based diets are well planned to include foods that are rich in these nutrients and methods such as soaking, sprouting, and fermentation to increase nutrient bioavailability (139).

The nutritional challenges may be particularly significant for certain groups, such as pregnant and lactating women, children, and the elderly. For example, a deficiency in iodine and omega-3 fatty acids during pregnancy may impact neurodevelopment in the fetus, and a deficiency in calcium and vitamin D may result in low bone mineral density in later life. Furthermore, highly processed plant-based diets, which are becoming more common in modern food systems, may be high in sodium, refined carbohydrates, and saturated fat, and low in essential vitamins and minerals. Therefore, the quality of dietary intake in a plant-based diet is a significant factor in influencing health outcomes. A focus on whole plant foods, such as legumes, whole grains, fruits, vegetables, nuts, and seeds, in addition to nutrient monitoring and supplementation, is crucial in ensuring the nutritional adequacy and health benefits of a plant-based diet (13).

### 7.2 Behavioral change, cultural identity, and food environments

To have a successful plant-based dietary transition, one must take into account that people’s food choices are influenced by factors beyond their conscious and deliberate rational decision-making especially when it comes to behavioral, psychological, and cultural issues (140). Research in behavioral economics has shown that food choices are largely influenced by default environments, social norms, affective responses to foods, habitual consumption patterns, and cognitive shortcuts that operate automatically and are hence highly resistant to informational interventions (141). Culture and food choice go hand in hand as a lot of the times meat consumption has always been part of the celebration, hospitality, social bonding, and identity in many cultures around the world, and hence it is quite costly psychologically for people to switch from meat-based to plant-based diets even if they are motivated by some reasons such as health or environment (24, 136–138). So, besides information and motivation, behavior change interventions need to be supported by interventions that modify the structural environments making plant-based choices default, socially expected, and economically rational (142). Some evidence-based behavior change strategies are serving plant-based options as default menu items in institutional food; cafe-traffic light labelling for simultaneously health and environmental; using social norm messaging campaign by citing up-to-date data on the trend of plant-based dietary adoption to persuade the audience; tax and subsidy policy measures aligning relative prices with health and environmental externalities; redesigning urban food environments by increasing the density and accessibility of outlets offering nutritious affordable plant food to the currently underserved communities (143).

### 7.3 Socioeconomic equity and structural barriers

Although there is overwhelming scientific evidence that plant-based dietary patterns are beneficial for health and environmental sustainability, there are, deep socioeconomic issues along with structural changes needed to allow for their democratic adoption (144). Ultra-processed foods dominate the diets of the poor and are the most spatially and economically accessible dietary options in many high-income countries and increasingly in lower-income countries as well. They have a high-energy content and great palatability due to the addition of numerous kinds of additives; they broadly marketed and their price per calorie is quite low compared to whole plant foods (84, 145). Agricultural policies in many countries maintain this inequity by subsidy programs that promote commodity grain production, which mostly used for animal feed and therefore supports animal protein production, while the diversified production of fruits, vegetables and legumes that would enable nutritionally optimal plant-based diets is just minimally supported (146). Food deserts areas that lack affordable fresh produce and healthy whole food, which usually inhabited by low-income, and minority populations in urban and rural settings are a direct structural manifestation of dietary inequity and simply cannot be solved by nutritional counselling (147). To effectively address the structural causes of dietary inequality, agricultural subsidies need to be reformed to support diverse plant food production, food retail (148). Distribution in underserved areas should be developed, food assistance programs expanded and improved nutritionally, plant-based nutrition literacy should be integrated into schools and communities, and fiscal measures including animal product taxes and plant-based subsidy programs should be used to make prices reflect health and sustainability (146, 147).

### 7.4 Food industry, product innovation, and regulatory frameworks

The plant-based diets industry is growing at a break-neck speed and a global market value of more than 77 billion have been projected (149). In the meantime, the shelf space of supermarkets and other retail outlets packed with a new and exciting range of plant-based meat alternatives, dairy substitutes, egg replacements, and processed plant-based convenience foods that are of great help to consumers who wish to cut down on animal products without compromising taste and convenience (139). Unfortunately, the nutritional heterogeneity of these commercial products is very significant such that while some of these products are made to have ridiculously good nutritional profiles and to provide meaningful amounts of protein, iron, calcium, zinc, and vitamin B12, many contain excessive sodium, tropical oil saturated fat, refined carbohydrate bases, lengthy ingredient lists filled with additives, and their long term health effects at habitual consumption levels are unknown (27, 150). Therefore, the commercial plant-based diets industry is a very complex regulatory challenge and the question is how to harness the brand, the popularity, and innovations in commercial plant-based products to help the consumers in the dietary transition whilst at the same time securing those products to contribute positively to population health outcomes instead of bringing harm (151, 152). The regulatory framework to meet this challenge must include mandatory comprehensive nutritional disclosure, evidence-based nutrient profiling standards specifically developed for plant-based products, restriction of misleading health and environmental marketing claims, minimum nutritional standards for plant-based products marketed as dietary alternatives to animal products, and investment in post-market surveillance research to evaluate the long-term health effects of novel plant-based diet ingredients and processing technologies (153).

### 7.5 Future perspectives

Future studies need to consider the following issues: the health outcomes of highly processed plant-based products, how to provide nutritional sufficiency for vulnerable groups, and the incorporation of plant-based diets into diverse food cultures. Policy strategies need to consider how to enhance access to affordable whole plant foods, how to promote sustainable food systems, and how to develop dietary recommendations that are compatible with health, sustainability, and culture (154). Most of the studies on plant-based diets are epidemiological observation, which are invaluable in revealing associations at the population level but are inherently subject to confounding by other health-conscious behaviors such as regular exercise, non-smoking, preventive visits to doctors, and higher socioeconomic status, which co-occur with making plant-based choices in the cohorts, and there are also limitations in dietary assessment accuracy when relying on self-report instruments (33, 34, 112, 155). Randomized controlled trials that are large enough and long enough to examine clinical hard endpoints such as cardiovascular events, cancer diagnosis, and mortality are very difficult, costly and time-consuming and yet are a very necessary complement to observational pieces of evidence for inference of causality and quantification of effect magnitude (156). Future research should focus on developing and validating objective dietary biomarker panels such as urinary polyphenol metabolites, fatty acid profiles, plasma carotenoids, and metabolomics-derived gut microbiome metabolites, which would allow for dietary exposure assessment to be more accurate and less biased than with conventional self-report instruments (157, 158).

Human gut microbiome is a very attractive area for mechanistic studies since we still do not fully understand the exact microbial pathways via which the components of plant-based diets have systemic health effects and inter-individual differences in microbiome composition and functional capacity are a major and quite under-recognized factor determining variations in dietary response among different individuals (159). Research in precision nutrition combining genomics, metabolomics, microbiomics, and continuous physiological monitoring (e.g., wearable devices tracking glucose or heart rate variability) could allow tailored plant-based dietary recommendations, which would fit individuals with diverse characteristics (160). At the same time there is a great need for population-level research to uncover the causes and impacts of socioeconomic disparities in plant-based dietary access, adoption, and health outcomes. So that the discourse between science and policy on plant-based dietary transitions does not only cater for the health and environmental objectives but also adequately addresses the equity dimension (161).

## 8 Conclusion

The review contrasted, and synthesized research from nutritional science, environmental sustainability, food systems analysis, and behavioral research. It concludes that plant-based diets are the better strategy to change human health and the environment as well. It is quite consistent as a well-planned plant-based diet can reduce the risk of developing all of the cardiovascular disease. It also provides protection against some types of cancers, have a diverse and metabolically active gut microbiota, reduce the risk of type 2 diabetes, causes less inflammation, oxidative stress and overall mortality. It is also equally convincing that the ecological footprint of plant-based dietary transitions is exceptionally overwhelming. Also, it points out that in contrast to animal-based foods there is much less greenhouse gas emissions, land use, freshwater usage, and ecological pollution footprint, when entire population switches their food to be in close proximity to plant-based diets. This way, 49–63% food system greenhouse gas emissions can be reduced and billions of hectares of arable land can be restored. Because of the formulation of circular food systems, it is possible to appreciate the systemic and structural excellence of phenomena that accompany the plant-based eating habits in terms of nutrient cycling, organic waste valorization, regenerative cultivation, and the incorporation of novel protein technologies. The lack of equitable access, behavioral and cultural change, regulation of quality of commercial products and methodological fortification of the evidence base are rather numerous issues that are to be solved yet they can be addressed in collaboration of the research, policy, industry and civil society. The empowerment of the potentials of the plant-based dietary patterns that are fair, nutritionally complete, culturally resonant, and regenerative of the environment is one of the most valuable common efforts to maintain human health and environmental integrity in the long term, today, and over generations.
